# Durable response from fibroblast growth factor receptor inhibition in intrahepatic cholangiocarcinoma terminated by metachronous acute myeloid leukemia: a case report

**DOI:** 10.1186/s13256-023-04231-2

**Published:** 2023-12-15

**Authors:** Andreas Edwin Juarso, Stefanie Entz, Florian Weissinger

**Affiliations:** 1 Department of Internal Medicine, Haematology/Oncology, Stem Cell Transplantation and Palliative Medicine, Evangelisches Klinikum Bethel, Schildescher Straße 99, 33611 Bielefeld, Germany; 2 Department of Internal Medicine and Gastroenterology, Evangelisches Klinikum Bethel, Schildescher Straße 99, 33611 Bielefeld, Germany

**Keywords:** Case report, Intrahepatic cholangiocarcinoma, Targeted tumor therapy, Fibroblast growth factor receptor, Secondary malignancy, Acute myeloid leukemia

## Abstract

**Introduction:**

Advances in the treatment of biliary tract cancer have been made possible through gains in genomic and epigenetic tumor understanding. The use of fibroblast growth factor receptor inhibitor has enabled significant clinical improvement in a specific group of patients with intrahepatic cholangiocarcinoma, some of whom with very durable responses.

**Case presentation:**

We present the case of a 69-year-old Caucasian patient with advanced intrahepatic cholangiocarcinoma who received the therapy with selective oral inhibitor of fibroblast growth factor receptor 1, 2, and 3 pemigatinib after multiple previous chemotherapies. This resulted in a durable stable disease condition for 15 months with good tolerability. The diagnosis of acute myeloid leukemia was an unanticipated serious adverse event, in which the impact of fibroblast growth factor receptor inhibition could not yet be determined due to inadequate data.

**Conclusions:**

It is still possible to achieve durable tumor response in advanced previously treated intrahepatic cholangiocarcinoma through targeted therapies. The prolonged progression free survival means that there could be an increased risk of secondary malignancy in this patient group, which necessitates diagnostic and therapeutic strategies.

## Introduction

Cholangiocarcinomas are aggressive tumors of the bile ducts, which have unfavorable prognosis despite multimodal therapies [1]. Since many patients remain asymptomatic in early stages, the diagnosis is usually confirmed only in advanced stages, resulting in poor prognosis. Relapses are common, even after curative surgical resection. The 5-year survival rate is about 7–20% [2]. Precision oncology, which is based on molecular targeted therapy, has been established in multiple cancer entities in recent years. This results in improvements in overall tumor response, progression free survival, and overall survival of some of the patients, even in advanced stages. Here we describe a patient with advanced intrahepatic cholangiocarcinoma, who had durable tumor response under the therapy with selective oral inhibitor of fibroblast growth factor receptor (FGFR) 1, 2, and 3 pemigatinib, which unfortunately was terminated upon the diagnosis of secondary acute myeloid leukemia.

## Clinical case presentation

A 69-year-old Caucasian patient with diabetes mellitus and hypercholesterolemia came to our hospital in June 2016 because of colic abdominal pain. Due to a suspected acute cholecystitis, a cholecystectomy was performed. Because visually altered liver tissues were observed during the procedure, a biopsy was also performed. The pathological report described malignant cells, which were most likely of hepatobiliary origin. A subsequent computed tomography (CT) scan revealed an approximately 50 mm tumor in the right lobe of the liver caudally. This prompted the resection of liver segment VI and a partial one of the segment V. In the resectate, the diagnosis of intrahepatic cholangiocarcinoma, with differentiation grade G2, was confirmed. Since it was an R0 resection of stage I tumor (pT1 according to international classification of diseases for oncology, 3rd edition), and there was no recommendation of adjuvant chemotherapy at that time, our patient went to follow-up-care and was observed for recurrence.

In November 2017, a tumor recurrence with extensive bilateral hepatic metastasis was detected during one of the follow-up evaluations. The patient was symptom-free at this time and the physical examination was unremarkable. He received palliative chemotherapy with cisplatin (25 mg/m^2^ on day 1 and 8) and gemcitabine (1000 mg/m^2^ on day 1 and 8) until disease progression in July 2019, after which the therapy was switched to 12 cycles of FOLFOX regimen (oxaliplatin 100 mg/m^2^, folic acid 400 mg/m^2^, and 5-fluorouracil (5-FU) 400 mg/m^2^ as bolus and 3000 mg/m^2^ as 48-h infusion every 2 weeks) until early February 2020. Because of further increases in the tumor marker CA 19–9 and the tumor size, the therapy was changed to FOLFIRI regiment (four cycles of irinotecan 180 mg/m^2^, folic acid 400 mg/m^2^, and 5-fluorouracil (5-FU) 400 mg/m^2^ as bolus and 2400 mg/m^2^ as 48-h-infusion every 2 weeks) until end of March 2020, which brought no tumor response.

In March 2020 a molecular evaluation of the original tumor material was performed using automated DNA extraction from Maxwell® RSC DNA FFPE Kit (Promega), DNA amplification through polymerase chain reaction and analysis through capillary electrophoresis (GenomeLab GeXP of Beckman Coulter), which identified an FGFR2 translocation/fusion (FGFR2-POC1B). A targeted therapy with pemigatinib, an oral inhibitor of FGFR1, 2, and 3, which was already accessed in the phase 2 clinical trial FIGHT-202 with positive results and granted US Food and Drugs Administration (FDA) approval, had not yet received the European Medicines Agency (EMA) approval at that time. We requested a “compassionate use” (expanded access) program for our patient, which allowed the early access use of pemigatinib for metastatic cholangiocarcinoma. The therapy was started in May 2020 with the dose of 13.5 mg/day for 2 weeks, followed by 1-week pause.

**Fig. 1:**
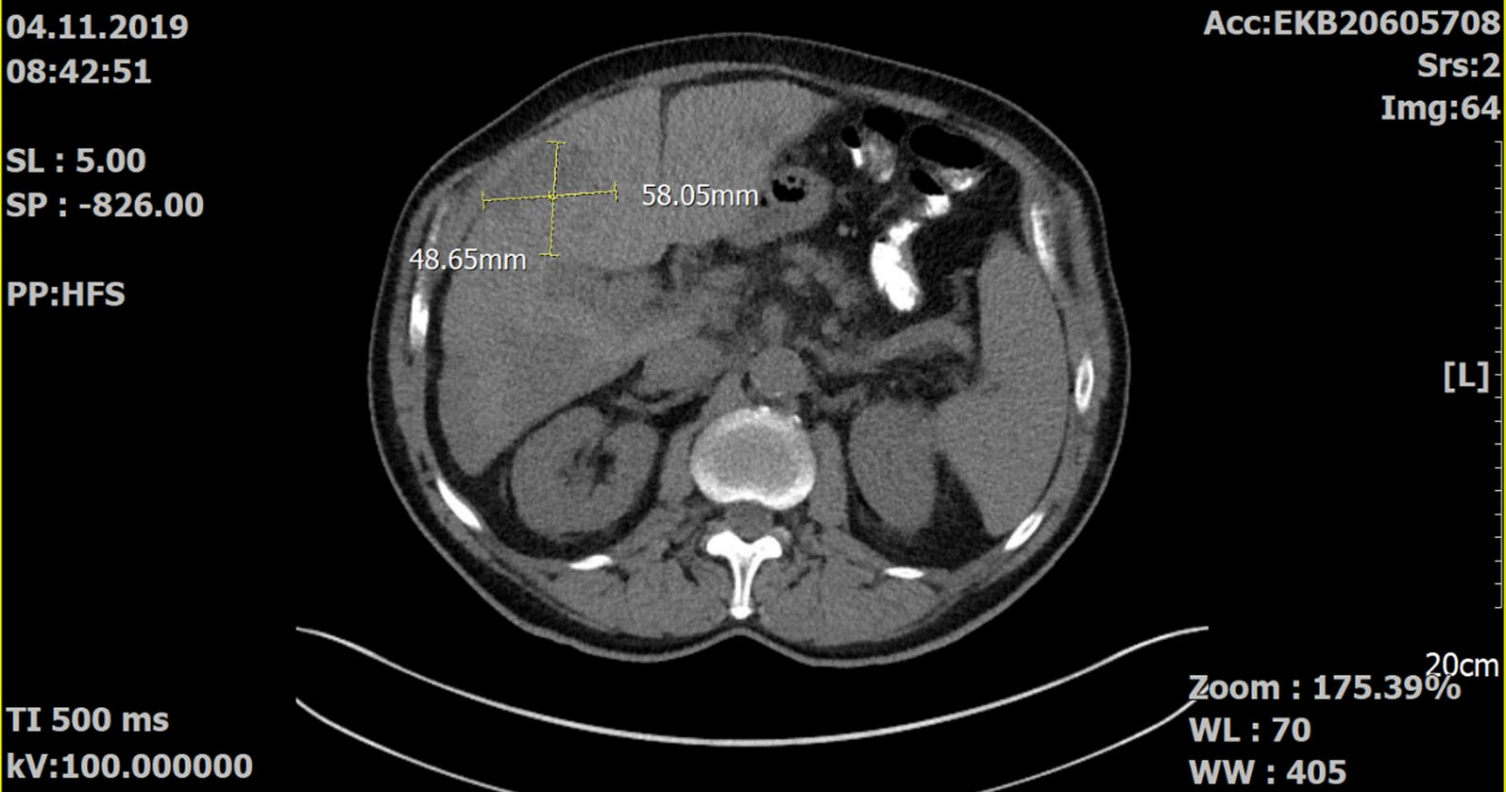
Selected CT images of the abdomen performed on November 2019 and August 2021

**Fig. 2:**
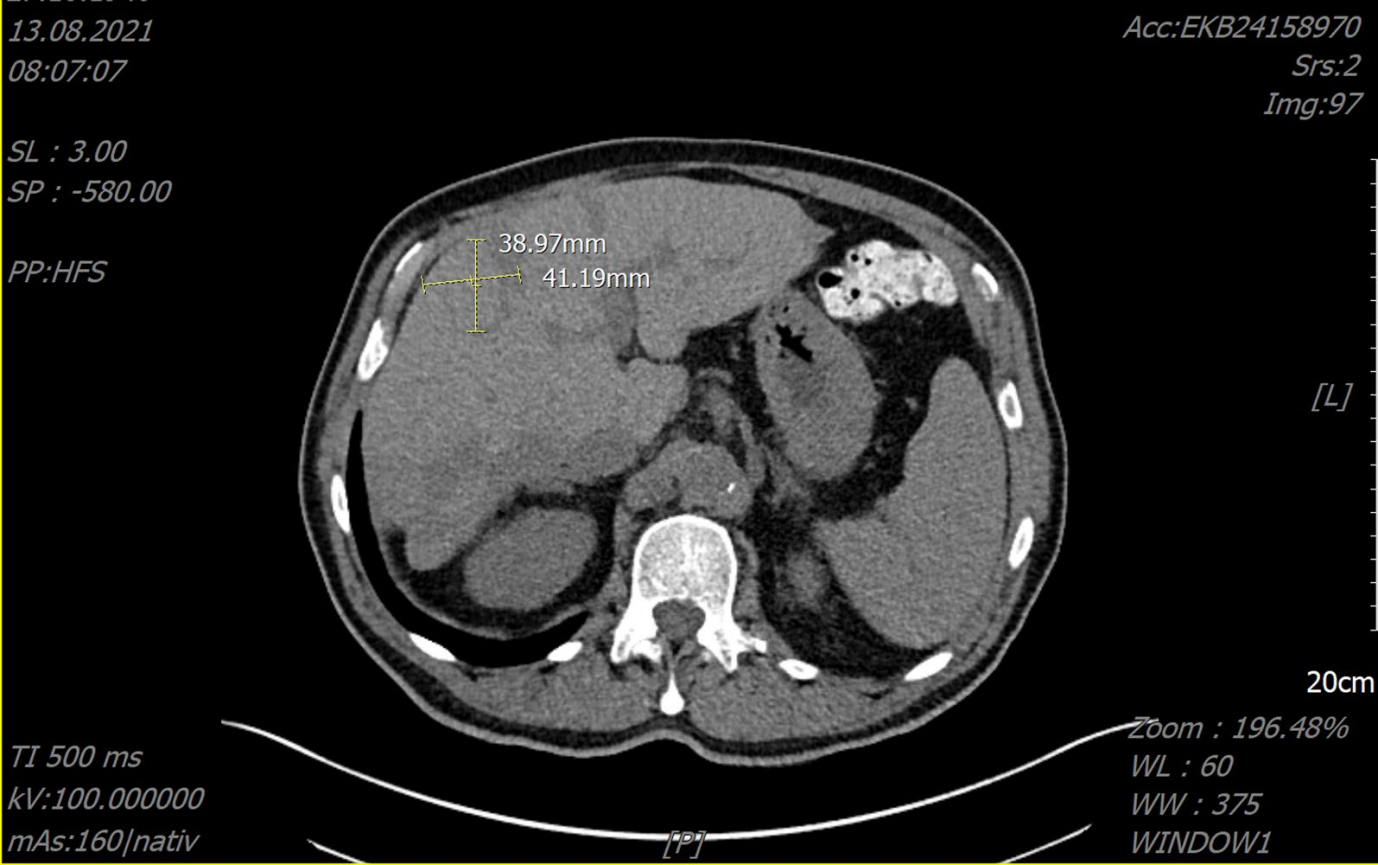
Selected CT images of the abdomen performed on November 2019 and August 2021

Our patient had a durable stable disease according to Response Evaluation Criteria in Solid Tumours, version 1.1 (RECIST), which lasted from May 2020 to August 2021 (Figs. 1, 2). During this therapy the patient had grade 3 anemia (Common Terminology Criteria for Adverse Events; CTCAE, version 5.0), which was treated with erythropoetin, and already preexisting grade 1–2 thrombocytopenia (CTCAE version 5.0) without the occurrence of relevant bleeding events (Table 1). After EMA approval in March 2021, pemigatinib could be prescribed without the expanded access program. Because of thrombocytopenia, the dose of pemigatinib was reduced from 13.5 mg to 9 mg/day for 2 weeks followed by 1-week pause.

**Table 1:** Laboratory data

Variable	Units	Reference	06/2016	11/2017	08/2019	04/2020	10/2020	06/2021	08/2021
Leukocyte	/nl	4.0–9.0	8.4	10.4	7.7	5.6	5.1	2.7	3.0
Erythrocyte	/pl	4.5–5.5	5.40	5.06	4.32	3.80	2.39	3.47	3.09
Hemoglobin	g/dl	14.0–18.0	16.3	15.4	13.1	12.3	7.1	10.8	8.8
Hematocrit	l/l	0.42–0.50	0.485	0.461	0.404	0.384	0.216	0.325	0.281
Mean corpuscular volume	fl	85–95	89.9	91.1	93.5	101.1	90.4	93.7	90.9
Mean corpuscular hemoglobin	pg	28–33	30.2	30.4	30.3	32.4	29.7	31.1	28.5
Mean corpuscular hemoglobin concentration	g/dl	32–36	33.6	33.4	32.4	32.0	32.9	33.2	31.3
Thrombocyte	/nl	150–400	148	183	182	84	65	51	24
Neutrophil	%	50–75	77.5	84.5					
Lymphocyte	%	25–40	14.9	9.2					
Monocyte	%	2–(6)12	4.4	5.2					
Eosinophil	%	2–4	1.4	0.5					
Basophil	%	0–1	0.8	0.6					
Blast	%								22
Promyelocyte	%								6
Myelocyte	%								6
Metamyelocyte	%								2
Bands	%								2
Segments	%								4
Lymphocyte	%								46
Monocyte	%								12
International normalized ratio		0.9–1.3	1.1	1.0					
Quick	%	70–120	91	107					
Partial thromboplastin time	Second	23–40	29	27					
Sodium	mmol/l	135–145	143	139	140	141	144	141	139
Potassium	mmol/l	3.6–5.3	4.23	4.60	4.25	4.50	4.30	4.29	4.43
Aspartate transaminase	U/l	< 50	16	22	36	16	24		22
Alanine transaminase	U/l	< 50	16	22	31	14	15		13
Cholinesterase	kU/l	4.6–11.5		10.90		8.47	6.96	7.81	7.14
Gamma-glutamyltransferase	U/l	< 60	62	59		182	29	36	46
Lipase	U/l	13–60							
Alkali phosphatase	U/l	40–130	92	93		165	81	128	110
Creatine kinase	U/l	< 190	69	83					
Lactate dehydrogenase	U/l	< 250	139	155	188	236	188	177	207
Protein	g/dl	6.6–8.7		6.9		7.2	7.0	7.1	7.3
Urea	mg/dl	10–50	22	36	48	34	69	49	50
Uric acid	mg/dl	3.4–7.0	6.2	6.1		7.4			
Creatinine	mg/dl	0.6–1.2	0.79	1.16	1.77	1.68	1.88	1.69	1.80
Phosphate	mg/dl	2.5–4.5				2.26		2.46	
Total bilirubin	mg/dl	< 1.0	0.5	0.3		0.3	0.6	0.5	0.5
C-reactive protein	mg/l	< 5	1.7	4.1		4.6	2.4	15	5.3
Ferritin	μg/l	30–400						1035	
CA19-9	U/ml	< 37	26	133		218	21	25	40
TSH	mIU/l	0.27–4.20		0.36			0.82

**Fig. 3:**
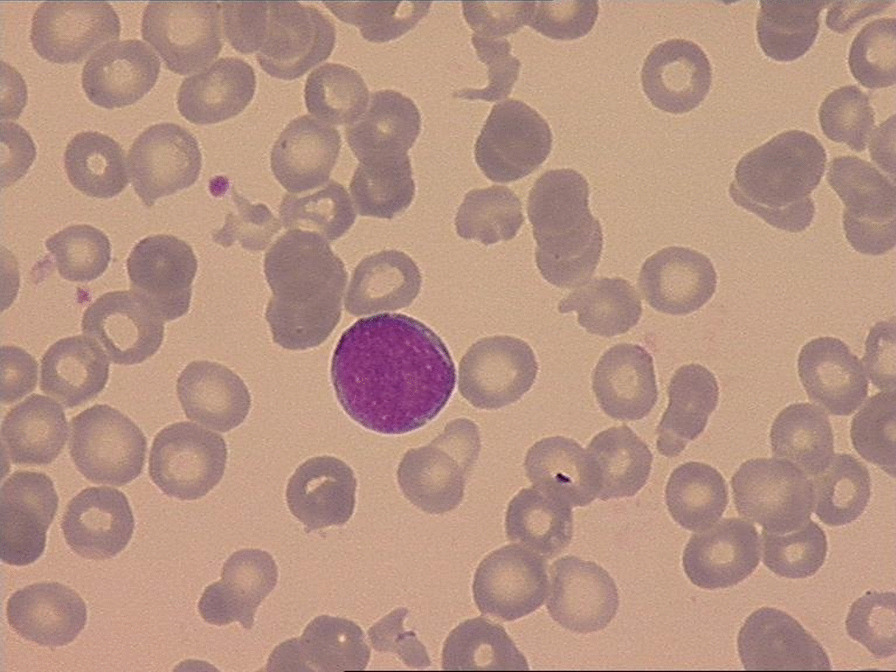
Peripheral blood and bone marrow aspirate smear in August 2021

**Fig. 4:**
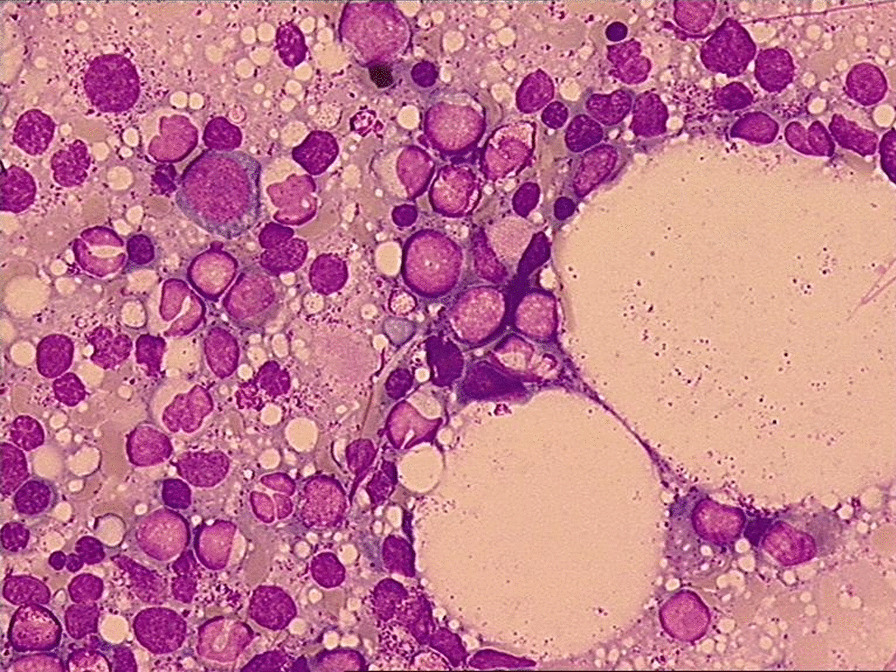
Peripheral blood and bone marrow aspirate smear in August 2021

Due to the worsening general weakness, thrombocytopenia, and anemia, the therapy with pemigatinib was stopped and we decided to perform a bone marrow biopsy in August 2021. The histological evaluation described a hypercellular bone marrow with dysplastic features of all three lineages, with blast cell percentage of 25%, confirming the diagnosis of acute leukemia, most likely due to underlying myelodysplastic syndrome (Figs. 3, 4). In the flow cytometry analysis there was an immature myeloid cell population with expression of myeloid antigen MPO, CD13, and CD33, corresponding to therapy-related myeloid neoplasms according to WHO classification. TP53 mutation in the molecular genetic test and complex karyotype involving rearrangements of chromosomes 4, 5, 7, 11, 12, 18, 19, and 20 were also identified. A diagnosis of therapy-related acute myeloid leukemia (t-AML) was made and he received a chemotherapy with azacitidine 75 mg/m^2^ (day 1–5) in September 2021. After a short clinical deterioration due to AML progression and pneumonia, he died in October 2021, 65 months after his initial diagnosis of intrahepatic cholangiocarcinoma.

As a part of pharmacovigilance standard procedure, we reported this case to the German Federal Institute for Drugs and Medical Devices, stating previous chemotherapy as the most likely cause of the acute myeloid leukemia. A separate report was also made to the medical product developer.

## Discussion

The fibroblast growth factor receptors 1–4 (FGFR 1–4) belong to the family of receptor tyrosine kinases (RTKs), which regulate cellular processes involving proliferation, cell cycle control, migration, and differentiation [3]. Aberrations in FGFR1–4 are found in 5–10% of human cancers, with increased frequency (10–30%) in urothelial cancer and intrahepatic cholangiocarcinoma [4]. The results of phase 2 study FIGHT-202 and FDA approval of pemigatinib in 2020 as the first FGFR1–3 inhibitor in the treatment of advanced intrahepatic cholangiocarcinoma with FGFR2 fusions or arrangements have led to improvements in the therapeutic strategies of this tumor entity [5, 6]. Since then, other FGFR inhibitors such as futibatinib, infigratinib, derazantinib, and erdafitinib have been shown to bring clinical benefits in terms of overall response and progression free survival, with relatively well-tolerated side effects [7–11].

There is no doubt that targeted therapy has brought significant benefit to our patient. Although pemigatinib was given as a fourth line therapy, it made a durable stable disease over 15 months possible. The clinical benefit in terms of the longer progression-free survival that our patient experienced could, however, bring another aspect into the light. In most guidelines, targeted therapies are currently recommended after more than one previous systemic therapies [12, 13]. Longer progression-free survival that results from these targeted therapies means that there could be an increased risk of secondary malignancy arising from previous chemotherapies a patient has received.

The diagnosis of metachronous therapy-related acute myeloid leukemia (t-AML) in our patient was an unanticipated serious adverse event. In our case, the acute myeloid leukemia (AML) most likely emerged as transformation from secondary chemotherapy related myelodysplastic syndrome, based on the multiple prior chemotherapies, characteristic myelodysplastic features in the bone marrow evaluation, and complex karyotype. The risk of t-AMLoccurence after chemotherapies, including platinum-based agents and topoisomerase I inhibitors (irinotecan), is well established, particularly in the first 5 years after initial cancer diagnosis [14]. The TP53 mutation and complex karyotype seen in our patient are already recognized as high-risk aberrations and are unfortunately associated with poor prognosis in AML [15, 16].

This case would have been straightforward, if new data had not been available. Besides having demonstrated efficacy in urothelial carcinoma with FGFR alterations [17], interim analysis has shown tumor agnostic efficacy of erdafitinib, an oral selective pan-FGFR tyrosine kinase inhibitor, in multiple other FGFR+ solid cancers [18]. Fusion genes involving FGFR1 are also known to be genetic driver in some hematologic malignancies with or without eosinophilia [19]. A case report from Kasbekar *et al*. described a durable complete hematologic and cytogenetic remission in a patient with a myeloid neoplasm with eosinophilia treated with futibatinib [20]. Furthermore, FGF2-FGFR1 signalling has been shown to be selectively activated in the bone marrows of patients with AML, favoring the survival of leukemia cells, progression, and therapy resistance, and that FGFR inhibition could reserve the stromal protection of leukemia cells and overcome resistance to kinase inhibitors [21]. In August 2022, the US FDA approved pemigatinib for treatment of relapsed or refractory myeloid/lymphoid neoplasms with FGFR1 rearrangement, based on the results of phase 2-FIGHT-203 Study (NCT03011372) that show a complete response of 75.8% in previously treated patients [22]. Another phase 1 trial evaluating pemigatinib after chemotherapy for the treatment of newly diagnosed acute myeloid leukemia (NCT04659616) is still recruiting.

To the best of our knowledge, there is currently no active clinical study involving FGFR inhibitor and therapy-related AML. In spite of this, we determined that it is important to make inquiry about the FGFR1 status of the leukemia cells in this case. The available cytogenetic analysis and fluorescence in situ hybridization (FISH) data shows a complex karyotype without involvement of chromosome 8, which is the location of FGFR1 (8p11.23) [23]. A consultation to our external partner laboratory was made to reperform the FISH analysis using the still available blood material to exclude cryptic FGFR1 rearrangement. It can be safely concluded that there is no evidence of FGFR1 rearrangements in the leukemia cells of our patient, a fact that is actually consistent with his treatment with an FGFR inhibitor. On the other hand, a positive result would have prompted further testing such as resistance mutation analysis. Nevertheless, it is not possible at the moment to deduce any positive or negative relationship between FGFR inhibitor and the occurrence of t-AML from this single patient case, and more data is certainly needed.

In the last few years more and more drugs, such as TRK, ALK, checkpoint, and BRAF, as well as FGFR, inhibitors have been or are being evaluated in basket clinical trials, with some of them already formally approved as tumor agnostic therapies [24]. Discordant outcomes are hypothetically possible in a patient with a primary and synchronous and/or consecutive metachronous secondary malignancies while on treatment with these drugs, which is certainly an area of interest and warrants investigations. This case highlights the need for further studies in the case of discordant outcomes of synchronous/metachronous malignancies in patients undergoing targeted tumor agnostic therapies, as well as the necessities of diagnostic work up, including resistance mutation evaluation, so as to improve therapeutic strategies for this specific patient group.

## Conclusion

Durable response in heavily pretreated advanced intrahepatic cholangiocarcinoma harboring FGFR2 alterations is possible with an FGFR inhibitor. However, the prolonged progression-free survival and overall survival resulting from this therapy means that there could be an increased risk of secondary malignancy in this patient group. Discordant outcome of different tumor entities in a patient treated with targeted tumor agnostic drugs presents a significant challenge and necessitates further studies to determine the appropriate diagnostic work up and therapeutic strategies for these patients.

## Data Availability

Laboratory data and images are available upon request.
